# Supplementary material for: Ca2+ Dynamics of Gap Junction Coupled and Uncoupled Deiters’ Cells in the Organ of Corti in Hearing BALB/c Mice
Source: Int J Mol Sci. 2023 Jul 4;24(13):11095. doi: 10.3390/ijms241311095 (PMC10342047; doi:10.3390/ijms241311095)
Supplement: Supplementary file 1 [file ijms-24-11095-s001.zip › ijms-2389936-supplementary.pdf]

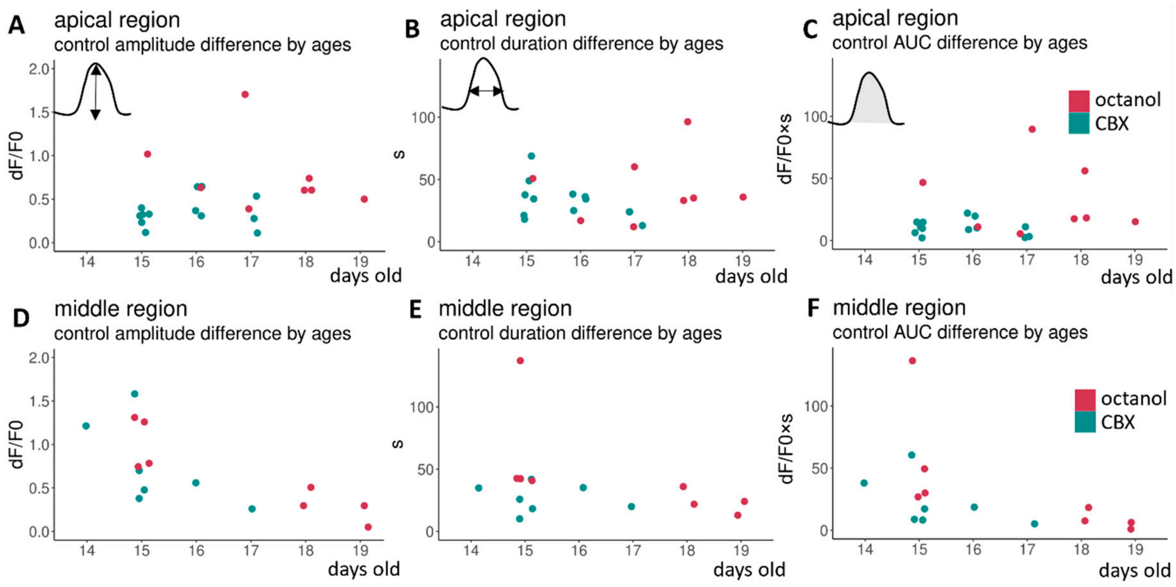

Supplementary figure S1. Age distribution of the control experiments. During the analysis of the results, we detected a difference between the controls of the octanol and CBX treated cells. We analysed the age of the experimental animals, which could have influenced the results. According to a linear model (included the control group, the age of the animal and the location of the cell) we did not find any statistical difference between the control groups.

Supplementary table S1. Variations between controls in the two investigated regions. The control responses of octanol- and CBX-treated cells were not significantly, but visibly different. The most reliable model parameter combinations were also different between the turns. The original column contains the original parameters from De Pittá, 2009. Parameters which were not changed in our models: rate of leakage (r_L = 0.11 s^{-1}), the ratio between the cytosol and ER volume (c_1 = 0.185) and the parameters of IP_3 receptor activation (a_2 = 0.2 $\mu M^{-1}.s^{-1}$; d_1 = 0.13 μM; d_2 = 1.049 μM; d_3 = 0.9434 μM; d_5 = 0.08234 μM)

	**Apical turns**		**Middle turns**		
	**controls of octanol**	**controls of CBX**	**controls of octanol**	**controls of CBX**	**original**
c_0 (μM)	4	1	1	2	2
κ_δ (μM)	2	1.1	1	1.5	1.5
K_3 (μM)	1.1	1.3	1.2	0.9	1
K_π (μM)	0.6	0.3	0.8	0.8	0.6
K_D (μM)	0.3	0.6	0.4	1	0.7
K_{ER} (μM)	0.05	0.05	0.11	0.07	0.1
K_P (μM)	13	12	13	14	10
$K_{PLC\delta}$ (μM)	0.09	0.15	0.13	0.09	0.1
K_R (μM)	1	1.3	1	2.1	1.3
$\overline{r_{5P}}$ ($1s^{-1}$)	0.03	0.07	0.02	0.09	0.04
r_C ($1\ s^{-1}$)	2	2	5	1	6

$\overline{v_{3K}}$ (μM·s^{-1})	4	3	1	3	2
$\overline{v_{\beta}}$ (μM·s^{-1})	0.4	0.4	0.1	0.3	0.2
$\overline{v_{\delta}}$ (μM·s^{-1})	0.02	0.03	0.02	0.05	0.02
v_{ER} (μM·s^{-1})	1.5	0.8	1.3	1.5	0.9
r_{P2X} (μM·s^{-1})	0.045	0.015	0.075	0.035	
v_{PMCA} (μM·s^{-1})	12.3	5.1	5.9	8.5	new variables
K_{PMCA} (μM)	2.75	2.22	2.03	2.42	
c_i (μM)	0.1	0.05	0.09	0.05	
h	0.8	0.8	0.9	0.9	
[IP_3] (μM)	0.3	0.1	0.5	0.5	
number of good fitted values (14)	11	13	11	13	

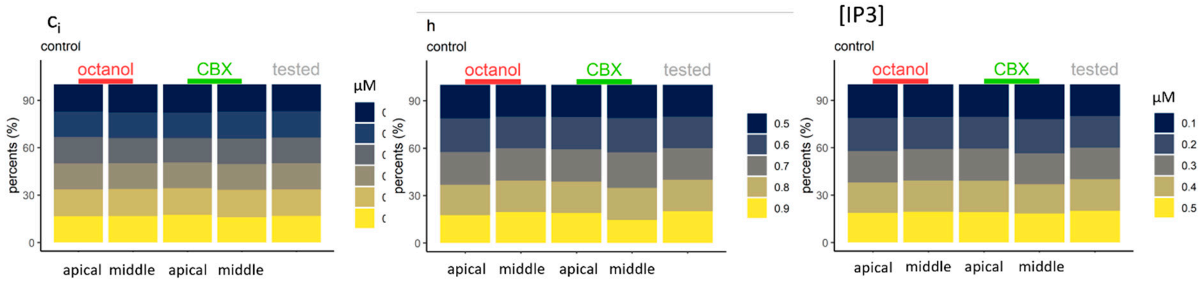

Supplementary figure S2. Ratio of the accepted initial values. A deeper analysis of the model parameters showed that the initial values did not influence the model success in fitting the data. All three initial values (Ca, h, IP3) are represented in the same ratio or in a ratio similar to that where they were tested.

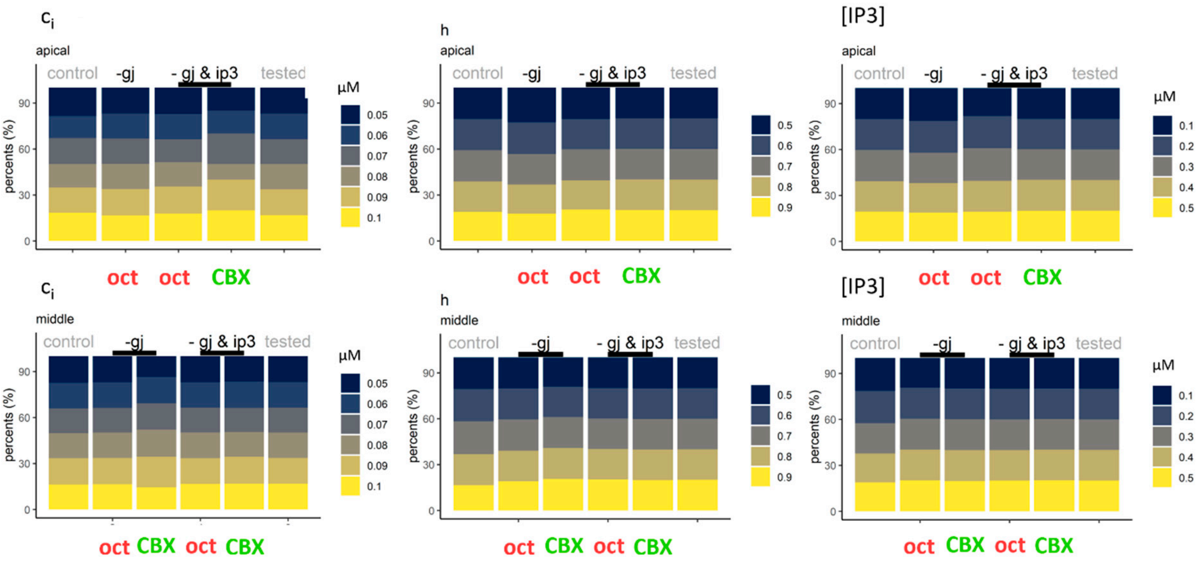

Supplementary figure S3. Ratio of the accepted initial values in the differently treated cells. Neither the removal of the gap junctional part (deep yellow) nor the removal of both gap junction and IP_3 parts (light yellow) of the model changed the initial conditions in the apical (upper row) or the middle (lower row) turns. This emphasize that they are not critical parameters in the model.

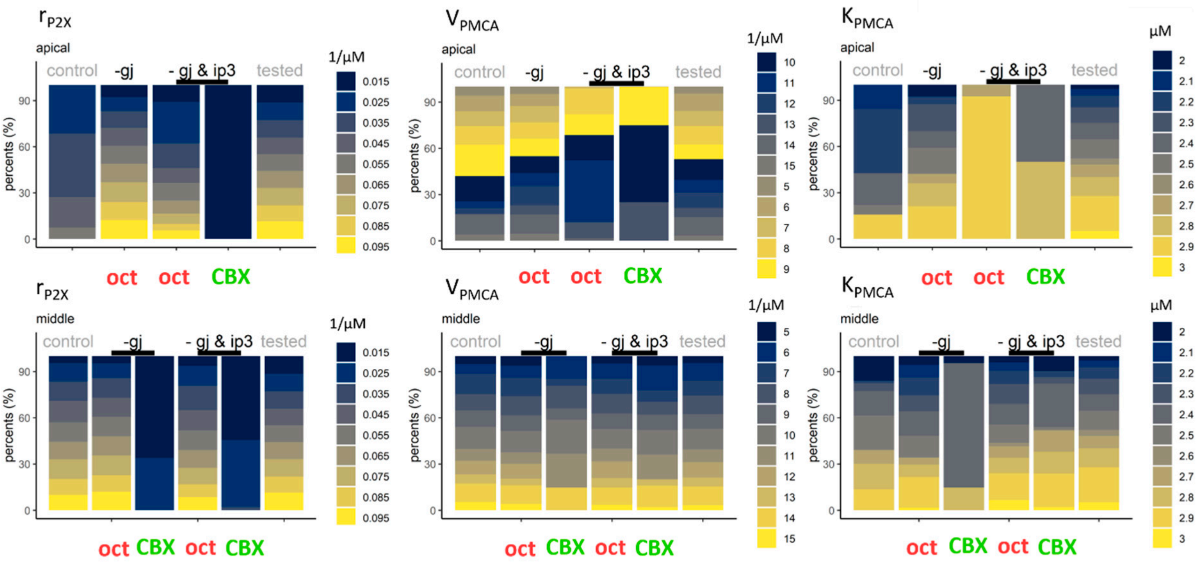

Supplementary figure S4. Parameters involved in Ca^{2+} movement through the plasmamembrane. P2X receptors activity highly influencing the CBX treated cells – only lower (0.015-0.025 1/uM) values were accepted in case of CBX treatment. PMCA pump parameters do not seem to be a limiting parameters in the middle region as their distribution is similar to the tested distribution. However, in the apical region in the drug treated cases higher V_{PMCA} parameters dominated. K_{PMCA} in the gap junction blocked group took lower values than in the gap junction and IP_3 pathway blocked groups.

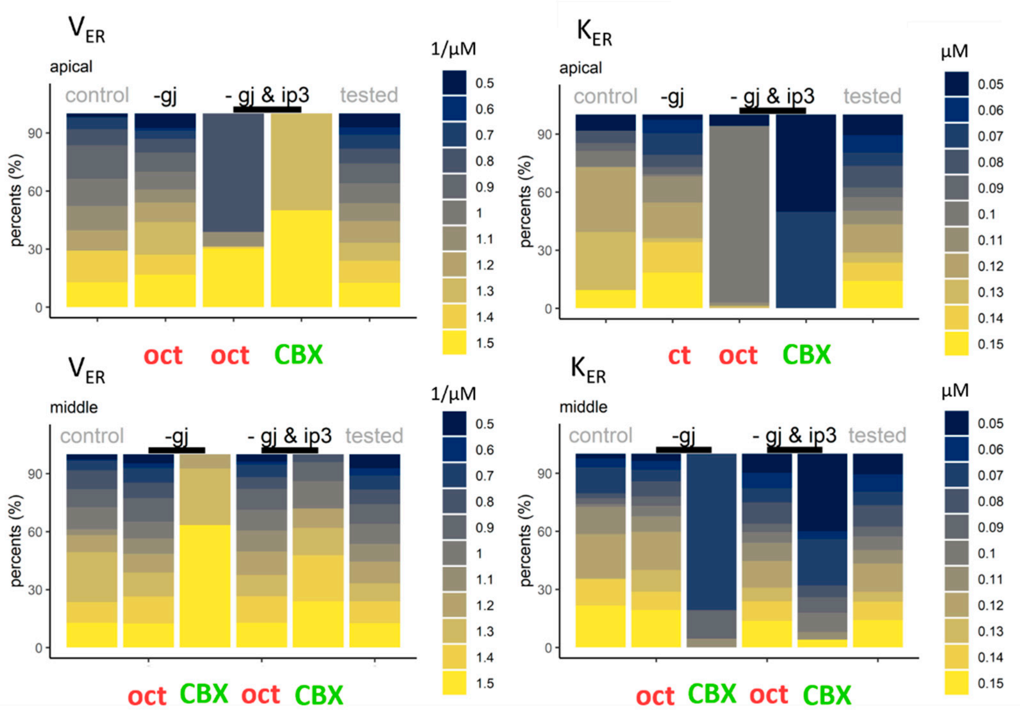

Supplementary figure S5. SERCA parameters. In the treated cells, when gap junction and IP$_3$ pathway blocking were hypothesised, the rate of the Ca^{2+} induced Ca^{2+} release was removed from the model and only the SERCA pump parameters were included. The velocity parameter of the pump (v_{ER}) is not a limiting parameter in case of the simply gap junction block (with octanol) as the accepted distribution of the values are similar to the tested. However, in case of the gap junction and IP$_3$ blocking in CBX treated cells it is a highly limiting factor – only 1.3-1.5 1/uM values were accepted. In case of octanol only 3 values were accepted, but these are covering the whole tested interval. In the middle region the CBX treated cells mostly took higher values,, while the octanol treated cells do not seem to be limited by this parameters. The Ca^{2+} affinity of the SERCA (K_{ER}) in the apical region, in case of the gap junction and IP$_3$ pathway blocking model is limited to the lower values, while in the middle region the CBX treatments accepted similarly the lower values.

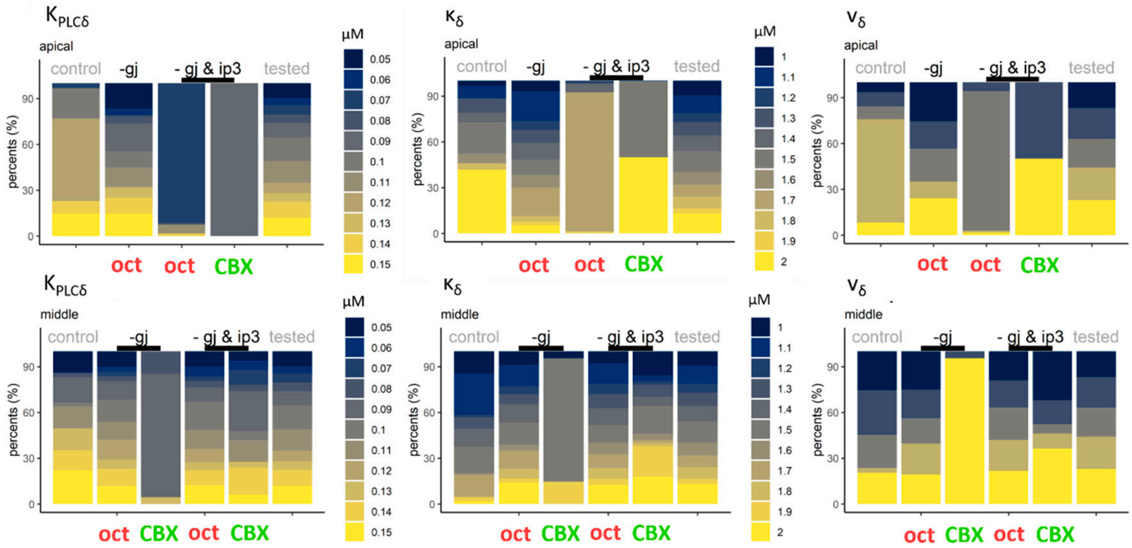

Supplementary figure S6. Parameters of the enzymes involved in the IP_3 generation. PLCδ activity parameters should not influence the models where the IP3 pathway are not involved. However, the in the apical region $K_{PLC\delta}$ took lower, κ_δ and v_δ higher values in these groups. In the middle region the PLCδ seems not to limit these groups, but has limiting effect in case of CBX treated cells, where only the gap junction blockade is hypothesised. In this group the $K_{PLC\delta}$ took the value 0.09 uM, κ_δ 1.5 uM and v_δ 0.05 values in most of the cases.

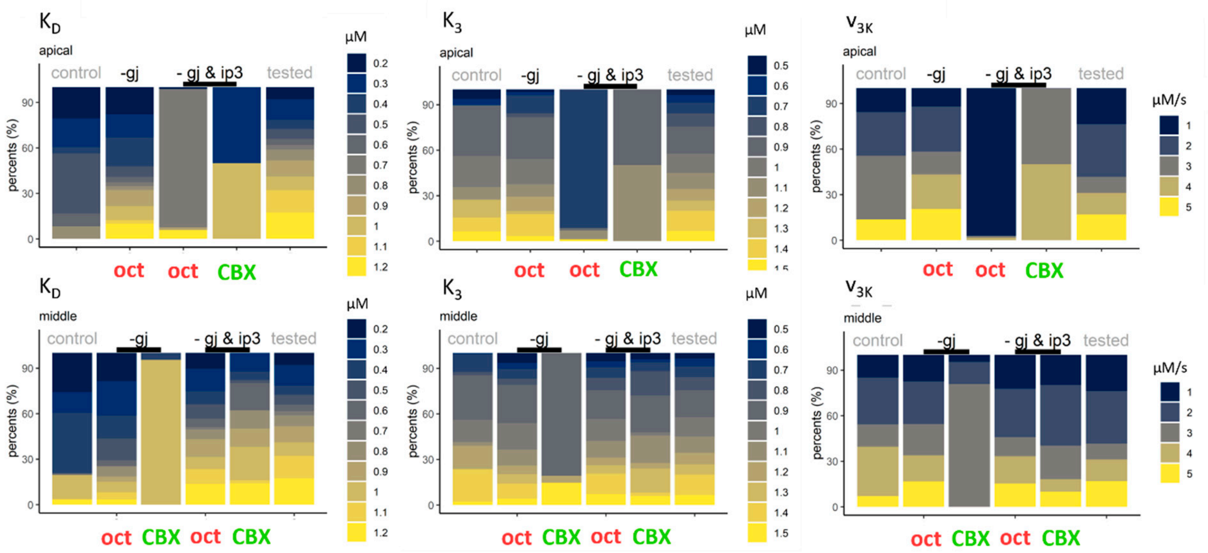

Supplementary figure S7. Parameters for inositol-3-kinases. Inositol-3-kinases are responsible to the degradation of IP_3, and because of this we did not expect to be limiting parameters when the IP_3 pathway are excluded. However, in the apical region these groups were differentiated from the tested parameter distribution: K_D took higher values, K_3 and v_{3K} lower values. In the middle region only the gap junction excluded CBX treated cells were different from the tested values. In this group the most prevalent values were: K_D = 1 uM, K_3 = 1 uM and v_{3K} = 3 uM/s.

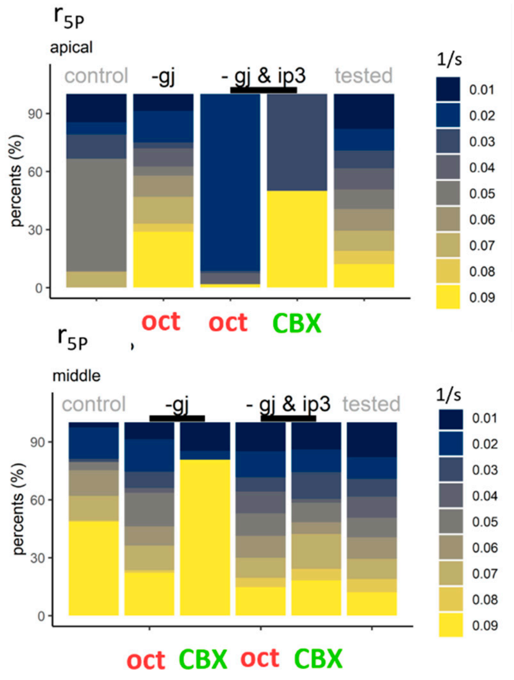

Supplementary figure S8. Parameters of 5-phosphatases. 5-phosphateases are involved in the degradation of IP3. Because of this we did not expect it be a limiting factor in treatment when IP_3 pathway is excluded. However, in the apical region the gap junction and IP_3 pathway blocked groups it seems to be a limiting parameter, more prevalently took lower values. In the middle region this groups were similar as the tested parameter distribution, but the gap junction blocked group this enzyme parameter seems to be limiting: in the CBX treated cells mostly 0.09 1/s value was accepted.
